# The Role of Tricho-Rhino-Phalangeal Syndrome (TRPS) 1 in Apoptosis during Embryonic Development and Tumor Progression

**DOI:** 10.3390/cells2030496

**Published:** 2013-06-27

**Authors:** Yujing Sun, Ting Gui, Aiko Shimokado, Yasuteru Muragaki

**Affiliations:** First Department of Pathology, Wakayama Medical University School of Medicine, 811-1 Kimiidera, Wakayama 641-0012, Japan

**Keywords:** Trps1, apoptosis, development, tumorigenesis

## Abstract

TRPS1 is a GATA-type transcription factor that is closely related to human tricho-rhino-phalangeal syndrome (TRPS) types I and III, variants of an autosomal dominant skeletal disorder. During embryonic development, Trps1 represses Sox9 expression and regulates Wnt signaling pathways that determine the number of hair follicles and their normal morphogenesis. In the growth plate, Trps1 regulates chondrocytes condensation, proliferation, and maturation and phalangeal joint formation by functioning downstream of Gdf5 signaling and by targeting at Pthrp, Stat3 and Runx2. Also, Trps1 protein directly interacts with an activated form of Gli3. In embryonic kidneys, Trps1 functions downstream of BMP7 promoting the mesenchymal-to-epithelial transition, and facilitating tubule morphogenesis and ureteric bud branching. Moreover, Trps1 has been found to be closely related to tumorigenesis, invasion, and metastasis in prostate and breast cancers. It is interesting to note that during the development of hair follicles, bones, and kidneys, mutations in Trps1 cause, either directly or through crosstalk with other regulators, a notable change in cell proliferation and cell death. In this review, we will summarize the most recent studies on Trps1 and seek to elucidate the role for Trps1 in apoptotic regulation.

## 1. Introduction

Mutations of the TRPS1 gene lead to tricho-rhino-phalangeal syndrome (TRPS) types I and III (TRPS I, OMIM 190350; TRPS III, OMIM 190351), which are variants of an autosomal dominant malformation syndrome characterized by craniofacial and skeletal abnormalities [1]. Craniofacial malformation includes sparse scalp hair, a bulbous tip of the nose, a long flat philtrum, a thin upper lip, and protruding ears. Skeletal abnormalities include cone-shaped epiphyses at the phalanges, hip malformations, and short stature. Momeni P., * et al*. have assigned TRPS1 to human chromosome 8q24, which is mapped on a proximal locus of the gene EXT1 [2].

The TRPS1 gene is approximately 260.5 kb in length and consists of seven exons. The Kozak consensus ATG translation start site is located in the third exon. Both human and mouse TRPS1 genes encode a large zinc-finger GATA-type nuclear protein comprising 1,281 amino acid residues and a calculated molecular mass of 141 kDa. There is 93% similarity between the TRPS1 proteins of the two species [3,4].

The protein domain of TRPS1 consists of nine zinc-finger domains, including a single GATA-type DNA binding domain flanked by two potential nuclear localization signals (NLS) and two *C-*terminal zinc-finger domains sharing a similarity of 55% with those of the Ikaros transcription factors [2]. TRPS1 belongs to the GATA transcription factor family. Unlike the other family members from GATA-1 to GATA-6, which have two C4-type GATA zinc-fingers and function as transcriptional activators [5], TRPS1 has only one C4-type zinc-finger and functions as a transcription repressor. An intact GATA zinc-finger is indispensable for DNA-binding, whereas the repressive activity of TRPS1 depends on the two *C*-terminal Ikaros-like zinc-finger domains. Truncated TRPS1 without 119 of the residues in the *C*-terminal (C119) fails to repress GATA4-activated transcription while a fusion protein GATA4 + C119 is exerting repressive activity. The two *C-*terminal zinc-finger domains of the Ikaros family have been reported to be involved in protein-protein interactions [6] through which Ikaros binds to several repressor proteins, including CtBP [7], Sin3 [8], and Brg1 [9]. After translocating to the nucleus, TRPS1 binds to the consensus GATA sequences in the promoter regions of target genes by its C4-type zinc-finger and suppresses transcription through the protein-protein interactions of its Ikaros-like domain by forming heterodimers with other transcriptional repressive factors [4]. The use of yeast 2-hybrid assays has demonstrated that TRPS1 can interact with the light chain 8 protein (LC8a) [10] and the ring finger protein RNF4 [11]. DNA binding assays and reporter studies revealed that binding TRPS1 with either LC8a or RNF4 diminishes the interaction of TRPS1 with GATA consensus sequences, consequently releasing the repressor activity of TRPS1. Recently, Trps1 has been reported to bind to the promoters of several Wnt inhibitors including Wif1, Apcdd1, and Dkk4, activating their transcription [12]. This adds the role of transcriptional activator to the established role of Trps1 as a transcription repressor. 

The function of Trps1 is studied by tracing Trps1 expression during embryonic development in mice and by morphogenesis studies in Trps1-deficient mice. Trps1 is expressed in a dynamic pattern with strict spatio-temporal regulation during mouse embryonic development [13]. Trps1 mRNA is detected prior to E7.5 with peak levels at around E11.5. From E12.5 to E14.5, Trps1 expression is intense in the facial region and pharyngeal arches, including the joints of limbs, maxilla, mandible snout, prospective phalanges, and hair follicles [4,13,14]. Trps1 expression is also detected in the developing lungs, gut, kidneys, and mesonephric ducts [13,15]. Both Trps1-null and Trps1^Δgt/Δgt^ mice die of respiratory failure soon after birth duo to abnormalities of their thoracic spines and ribs [14,16]. Trps1^−/−^ newborns have a prominently decreased number of hair follicles; skeletal malformation, including shortened bones in the limbs, facial abnormalities, and thoracic defects; poorly inflated lungs; and ill-developed kidneys [14,15,16,17]. The phenotypes found in Trps1-deficient mice mirror those found in TRPS patients, suggesting the indispensable role of TRPS1 in embryonic development.

Before Trps1 was assigned to be the causative gene whose mutation leads to tricho-rhino-phalangeal syndrome, GC79 (the previous name for TRPS1) was found to be one of the differentially expressed genes between androgen-dependent and independent prostate cancers [3]. Subsequently, Trps1 was identified to be the most prevalently expressed gene in breast tumors [18]. Collective evidence shows that Trps1 may play a protective role in preventing tumor growth, invasion, and metastasis by promoting apoptosis and counteracting epithelial-mesenchymal transitions [3,19,20]. Recently, it has been reported that patients with mutations in the TRPS1 gene have increased susceptibility to tumorigenesis [21].

Apoptosis, also known as programmed cell death, is crucial in embryonic development, organ morphogenesis, normal tissue cycling, and tumorigenesis. Tprs1 has been shown to play pivotal roles in the development of bone [14], distal phalangeal joints [22], the temporomandibular joint [17], and hair follicles [23], as well as in tumor progression [24], by regulating key factors that participate in signaling pathways controlling cell proliferation and apoptosis. Here, we will briefly review Trps1 and how it exerts its function in embryonic development and tumor progression by interfering with cell proliferation and death. 

## 2. The Role of Trps1 in Developing Hair Follicles, Bone, and Kidney

TRPS1 is indispensible in normal hair follicle morphogenesis. Trps1 protein first appears in the dorsal epidermis in the undifferentiated epithelium at E14.0, whose expression is transient and diffuse. Later, at E15.5, Trps1 protein is observed in the dermal condensate; by E17.5, Trps1 protein is restricted in dermal papilla cells and the mesenchymal cells surrounding the hair pegs and underlying the epidermis [25]. Trps1^Δgt/Δgt^ neonates have an almost 50% reduction in the number of pelage follicles in the dorsal skin and completely lack vibrissa follicles [16]. Developing Trps1^Δgt/Δgt^ vibrissa follicles are irregularly spaced with reduced number and size, compared to their wild-type littermates at E16.5. Just after the initiation of epithelial peg downgrowth, the further development ceases and undergoes subsequent degeneration [12]. A marked increase in proliferation throughout Trps1^Δgt/Δgt^ mutant vibrissa follicles has been demonstrated by immunostaining for Ki67. Elevated Sox9 expression in both mRNA and protein levels is suggested to be the underlying mechanism [23]. Sox9 has been well demonstrated in a number of studies with regard to its pro-survival role during chondrogenesis [26] and hair follicle development [27], and recently it has been reported to have oncogenic potential [28]. Trps1 represses Sox9 transcription by direct promoter binding [23]. So it is likely that enhanced Sox9 expression due to a loss of Trps1 leads to active cell proliferation.

Microarray hybridization analysis between wild-type and Trps1^Δgt/Δgt^ whisker pad in early stage of vibrissae development (E12.5) identifies number of transcription factors and Wnt inhibitors are downregulated and several extracellular matrix proteins are upregulated in Trps1^Δgt/Δgt^ whisker pad. Wnt signaling is upregulated in epithelial cells of the placode in mutant follicles [12]. Trps1 is likely to regulate the normal early vibrissa follicle organogenesis by orchestrating a wide range of gene expression. 

Trps1^−/−^ newborn mice have shortened long bones and incompletely formed phalangeal joints. Histological examination of Trps1^−/−^ newborn tibiae revels an expanded length of the proliferative zone with a reduced chondrocyte density and a normal area of the hypertrophic zone in the growth plate. The differentiation of Trps1^−/−^ chondrocytes is normal with non-altered expression of Col II, Col X, and Ihh signaling [14]. Different from Trps1^−/−^ mice, in Trps1^Δgt/Δgt^ mice that have an in-frame mutation in GATA-DNA binding domain, both elongated proliferative and hypertrophic zone are observed, with enhanced Ihh signaling, and elevated Col X and Runx2 expression. Trps1^−/−^ mice represent the animal model of human TRPS type I, whereas Trps1^Δgt/Δgt^ mice reflect the TRPS type III, which are similar to TRPS type I except with severe generalized shortening of all phalanges and metacarpals [29,30]. Although the underlying mechanism is not clear, it is speculated to be due to gain of function of a Trps1Δgt allele, in addition to the loss of DNA-binding and repressive activity [31]. Apoptosis in hypertrophic chondrocytes is greatly inhibited in mutant growth plates compared to wild-type littermates. An *in vitro* study has demonstrated that cultured primary Trps1^−/−^ chondrocytes are more resistant to Jo2-induced apoptosis than wild-type chondrocytes. Reporter and ChIP assays have revealed that Trps1 inhibits Stat3 transcription by directly binding to GATA consensus sequences in the Stat3 promoter [14]. Stat3 has been reported to directly increase Bcl-2 and support hepatocyte survival, which is an important anti-apoptotic regulator [32]. Thus, it is possible that Trps1 exerts its pro-apoptotic functions by indirectly repressing Bcl-2 expression. Indeed, Trps1^−/−^ chondrocytes showed higher Bcl-2 expression after death-induction by Jo2. Another study has demonstrated that Trps1 represses PTHrP expression by direct promoter binding, contributing to an expanded proliferative zone in Trps1^−/−^ growth plate [33]. PTHrP inhibits major death pathways by blocking signaling via p53, death receptors, and mitochondria in tumor cells, so we can expect Trps1 to play a role in pro-apoptosis by counteracting PTHrP [34,35]. 

The development of the mammalian kidney is the result of a programmed reciprocal induction between ureteric buds (UBs) and the metanephric mesenchyme [36]. During kidney development, Trps1 is expressed in the ureteric buds, cap mesenchyme, and renal vesicles. Trps1^−/−^ developing kidneys exhibit fewer tubules and glomeruli and have expanded interstitium compared to wild-type mice [15] and defects in UB branching are also observed, which are possibly due to the abnormal mesenchyme and ineffective reciprocal induction [37]. Several studies have done to reveal the role of Trps1 in developing kidneys. Trps1 acts downstream of Bmp7 via the Bmp7/p38 MAPK/Trps1 pathway mediating Bmp7-induced mesenchymal-to-epithelial transition which lead to formation of tubules and glomeruli and is essential for normal renal development [15]. The loss of Trps1 leads to increased activation of TGF-β/Smad3 signaling [38], which is also observed in developing kidneys [39]. Expression patterns of several genes associated with the TGF-β/Smad3 signaling pathway, such as Rb1cc1, Arkadia, Smurf2, Smad7, and c-ski are altered in Trps1^−/−^ mice during kidney morphogenesis. Previous studies on TGF-β1 during tubulomorphogenesis have indicated that TGF-β1 functions as a negative regulator in ureteric bud development. The addition of TGF-β1 into culture medium of the ureteric buds leads to a decrease in overall size, branching numbers, and BrdU-positive UB tip cells and an increase in the thickness of UB stalks and the number of cells undergoing apoptosis [39,40,41], whereas the exogenous addition of SIS3, a Smad3 inhibitor, was able to restore Trps1^−/−^ UB branching [39]. 

Multiple genes are required for branching morphogenesis, however, the molecular mechanism remains largely unclear. Many studies suggest that active cell proliferation and inhibited apoptosis is the key feature of normal kidney development [42,43]. A number of genes, such as Pax2 [43], Bcl-2 [44], Mdm2 [45], and Mmp9 [46], have been identified as a fate-determining gene in kidney morphogenesis by regulating cell apoptosis. Mutations in these genes cause fulminant apoptosis in UB cells and subsequent dramatic decrease in UB branching. In accordance with this hypothesis, Trps1^−/−^ developing kidneys manifest low levels of cell proliferation and boosted apoptosis [39]. Hence we speculate that Trps1 plays a role in maintaining cell proliferation and counteract apoptosis in normal kidney morphogenesis. 

## 3. TRPS1 Promotes Apoptosis and Counteracts Metastasis in Tumor Cells

TRPS1 was first discovered as one of the differentially expressed genes between androgen-dependent (LNCaP-FGC) and androgen-independent (LNCaP-LNO) prostate cancer cell lines [47]. TRPS1 protein was found to co-express with androgen receptors and PSA in androgen-dependent prostate cancers. TRPS1 is androgen-repressible in androgen-dependent, but not in androgen-independent, prostate cancers. Recently, TRPS1 mRNA was also detected in human breast cancer xenografts [48].

TRPS1 has begun to attract wide attention as an important regulator of apoptosis, as it has been found that TRPS1 protein expression is androgen-suppressive in androgen-dependent (LNCaP-FGC) prostate cancer cells but not in androgen-independent (LNCaP-LNO) prostate cancer cells [47]. Adding androgen to the culture medium promotes tumor cell growth and simultaneously represses TRPS1 mRNA expression [47]. In a regressing rat ventral prostate, castration-induced androgen withdrawal causes prostate cell apoptosis, a notable increase in Trps1 mRNA levels, and changes in a set of oxidative stress-related genes [3,49,50]. On the other hand, over expression of the TRPS1 gene by transiently transfecting TRPS1-encoding vectors leads to a dramatic increase in the apoptotic index of both COS-1 cells and LNCaP prostate cancer cells [3]. Proteomic analysis of androgen-independent DU145 prostate cancer cells that do not express TRPS1 and of genetically engineered DU145 cells that stably express recombinant TRPS1 have demonstrated that TRPS1 suppresses the protein expression of certain antioxidants, including superoxide dismutase, protein disulfide isomerase A3 precursor, endoplasmin precursor, and annexin A2 [51]. These studies suggest that the involvement of TRPS1 in apoptosis is to occur by perturbing the intracellular reduction-oxidation balance. 

As previously discussed, in hair follicles of developing mice, Trps1 is able to activate several Wnt inhibitors, thus suppresses Wnt signaling [12]. Although there is no studies to investigate the relation between Trps1 and Wnt signaling in tumors cells, the deregulation of β-catenin activation do leads to tumors, such as colon cancer, leukemia, hair follicle tumors, and a wide variety of solid tumors [52], and this oncogenesis is at least partly related to elevated expressions of cyclin D1, c-myc, and the anti-apoptotic factor survivin [53,54,55]. Hence, it will be interesting to examine Trps1 expression in tumors that harbor up-regulated Wnt signaling. We speculate that Trps1 might play a role in tumorigenesis by regulating cell proliferation and cell death via interference with Wnt signaling. 

Based on a comprehensive differential gene expression screen, Trps1 has been revealed as one of the most prevalent genes that is specifically overexpressed in breast cancer [18]. A quantitative immunohistochemistry (qIHC) approach found that TRPS1 protein expression in breast cancers above a certain threshold was correlated with markedly improved overall survival [20]. Trps1 is a target gene of miR-221/222 in luminally originated breast cancer cells counteracting EMT that restrains tumor cells from metastasis [19]. Collectively, TRPS1 is considered to be related to a better clinical prognosis of breast cancer. 

## 4. Conclusion

An increasing number of studies are demonstrating that Trps1 is an indispensable regulator of embryonic development, growth plate regulation, and tumor morphology. Among these, a number of studies have made intriguing discoveries that hint at an apoptosis-regulating role for Trps1. As reviewed above, Trps1 regulates apoptosis either by directly repressing pro-survival factors, such as Sox9 and PTHrP, or by indirectly suppressing signaling pathways, such as Wnt and JAK-STAT signaling that favor cell survival. On the other hand, TGF-β1/Smad signaling is tightly regulated by Trps1 [38]. It has been reported that the incubation of cultured UB with TGF-β1 leads to enhanced apoptosis in UB tips. Thus, Trps1 may be perceived as an anti-apoptotic factor, a result consistent with our observation that during UB branching, Trps1^−/−^ UBs presents enhanced expression of TGF-β1 and apoptosis (Table 1).

**Table 1:** Target genes of tricho-rhino-phalangeal syndrome (Trps) 1, interaction and function of Trps1, the tissues where the interaction with Trps1 takes place, and the consequences of the interaction.

Target Genes	Interaction/function of Trps1	Tissues	Consequences of Trps1 deficiency	References
PTHrP	Direct promotor binding/repressive	Developing growth plate	Enlongated proliferative zone	[33]
Stat3	Direct promotor binding/repressive	Developing growth plate	Apoptosis resistance due to elevated Bcl-2	[14]
Sox9	Direct promotor binding/repressive	Developing vibrissae follicles	Increased cell proliferation	[23]
Wnt inhibitors	Direct promotor binding/activative	Developing vibrissae follicles	Enhanced Wnt signaling	[12]
Anti-oxidants	Unknown/repressive	Prostate cancer cells	Apoptosis resistance	[3,47,50]
TGF-β/Smad3 pathway	Unknown/repressive	Developing kidneys	Increased apoptosis	[38,39]

In conclusion, Trps1 may act as both pro- and anti-apoptosis regulators in the context of microenvironment. However, no research currently exists on how these changes in apoptosis take place. There is lack of direct evidence showing direct interaction between Trps1 and classical apoptotic regulators. It will be interesting to investigate these apoptotic pathways with delicate experimental designs to hopefully elucidate the exact role of Trps1 in the regulation of apoptosis.
